# Glucose-6-Phosphate Dehydrogenase Activity in Milk May Serve as a Non-Invasive Metabolic Biomarker of Energy Balance in Postpartum Dairy Cows

**DOI:** 10.3390/metabo13020312

**Published:** 2023-02-20

**Authors:** Ayelet Hod, Jayasimha Rayalu Daddam, Gitit Kra, Hadar Kamer, Yuri Portnick, Uzi Moallem, Maya Zachut

**Affiliations:** 1 Department of Ruminant Science, Institute of Animal Sciences, Volcani Institute, 68 HaMaccabim Road, Rishon LeZion 7505101, Israel; 2 Department of Animal Science, The Robert H. Smith Faculty of Agriculture, Food and Environment, The Hebrew University of Jerusalem, Rehovot 76100, Israel

**Keywords:** energy balance, glucose-6-phosphate-dehydrogenase, milk, dairy cows, fatty acid profile

## Abstract

Negative energy balance (EB) postpartum is associated with adverse outcomes in dairy cows; therefore, non-invasive biomarkers to measure EB are of particular interest. We determined whether specific metabolites, oxidative stress indicators, enzyme activity, and fatty acid (FA) profiles in milk can serve as indicators of negative EB. Forty-two multiparous Holstein dairy cows were divided at calving into 2 groups: one was milked 3 times daily and the other, twice a day for the first 30 d in milk (DIM). Cows were classified retrospectively as being in either negative EB (NEB, *n* = 19; the mean EB during the first 21 DIM were less than the overall median of −2.8 Mcal/d), or in positive EB (PEB, *n* = 21; the mean EB was ≥−2.8 Mcal/d). The daily milk yield, feed intake, and body weight were recorded individually. Blood samples were analyzed for metabolites and stress biomarkers. Milk samples were taken twice weekly from 5 to 45 DIM to analyze the milk solids, the FA profile, glucose, glucose-6-P (G6P), G6P-dehydrogenase (G6PDH) activity, malic and lactic acids, malondialdehyde (MDA), and oxygen radical antioxidant capacity (ORAC). The NEB cows produced 10.5% more milk, and consumed 7.6% less dry matter than the PEB cows. The plasma glucose concentration was greater and β-hydroxybutyrate was lower in the PEB vs. the NEB cows. The average concentrations of milk glucose, G6P, malic and lactic acids, and MDA did not differ between groups; however, the G6PDH activity was higher and ORAC tended to be higher in the milk of NEB vs. the PEB cows. The correlation between milk G6PDH activity and EB was significant (r = −0.39). The percentages of oleic acid and total unsaturated FA in milk were higher for the NEB vs. the PEB cows. These findings indicate that G6PDH activity in milk is associated with NEB and that it can serve as a non-invasive candidate biomarker of NEB in postpartum cows, that should be validated in future studies.

## 1. Introduction

During the transition from late gestation to lactation, high-yielding dairy cows experience a sudden shift in their energy demand for milk production, which induces tissue mobilization and negative energy balance (NEB) [1,2]. Negative EB postpartum is associated with several adverse outcomes on cow health and performance [3,4,5]. Therefore, in recent years, there has been growing interest worldwide in establishing empirical non-invasive indicators of cow health and fitness [6]. Identifying biomarkers of NEB is of great importance to the dairy industry, since direct measurement of feed intake, which is required for calculating energy balance, is not feasible in a commercial setting. Xu et al. [7] used liquid chromatography-mass spectrometry and nuclear magnetic resonance techniques to identify metabolic changes in milk of early lactation cows. Importantly, they found that 15 metabolites were positively correlated with EB and 20 were negatively correlated with it, which could be attributed to the increased leakage of cellular content and the elevated synthesis and metabolism in epithelial cells during NEB.

Epithelial cells in the mammary gland do not synthesize glucose due to a lack of the enzyme glucose-6-phosphatase [8]. Therefore, milk glucose concentrations are dependent on glucose absorbed from the blood. Within the mammary gland, glucose is converted to glucose-6-phosphate (G6P), which is a central metabolite in the glycolytic pathway and is an intermediate compound during lactose synthesis; G6P participates in the initial steps of both glycolysis and the pentose phosphate pathway (PPP) [9]. The enzyme G6P-dehydrogenase (G6PDH) is the first enzyme in the PPP that converts G6P into 6-Phosphogluconolactone. In early lactation, milk glucose is first low and then gradually increases, whereas G6P in milk is high postpartum and then decreases in the milk of dairy cows [10,11]. Thus, in a study that involved a small number of cows, a significant correlation was found between G6PDH activity and G6P content in milk postpartum [11]. Based on these findings, we previously postulated that the balance between these biochemical pathways (glycolysis and PPP) within the mammary gland, which may be reflected in the concentrations of glucose, G6P, and G6PDH activity, is associated with the energetic and oxidative state of the cow, as a part of the homeostatic adaptation to NEB at the onset of lactation [11]. Therefore, these compounds could serve as biomarkers of cows’ physiological state postpartum [6]. Here, we aimed to validate and further examine several milk metabolites (glucose, G6P, malic acid, and lactic acid), G6PDH activity, markers of oxidative stress, and the fatty acid (FA) composition of milk in an intensive and comprehensive study with postpartum dairy cows, to determine the relationship between NEB and candidates for milk biomarkers in high-yielding dairy cows.

## 2. Materials and Methods

### 2.1. Cows and Experimental Procedures

The experimental protocol and procedures were approved by the Volcani Center Animal Care Committee (IL 637/16). The study was conducted at the experimental dairy farm of the Volcani Center in Rishon LeZion, Israel. A detailed account of the study procedures was published [12]. Briefly, 42 multiparous high-yielding Holstein cows were divided into 2 subgroups: 21 cows were milked 3 times a day (at 05:00, 13:00, and 20:00 h), and 21 cows were milked twice a day (at 07:00 and 19:00 h) until 30 days in milk (DIM); from 30 DIM, all cows were housed together and milked 3 times a day. From day 5 postpartum until 30 DIM, milk samples were taken twice a week (Monday and Thursday) from 2 consecutive milkings for cows that were milked twice daily, or from 3 consecutive milkings for cows that were milked thrice daily. We assumed that the different milking frequencies would influence the milk yield and intake, and consequently, EB, resulting in postpartum cows with varied EB. From 30 to 45 DIM, milk samples were taken from 3 consecutive milkings twice a week. Milk samples were analyzed for milk fat, protein, and lactose by infrared analysis (standard IDF 141C: 2000). The milk fat FA profile was determined using a Fourier transform mid-infrared spectrometer (Bentley FTS, Bentley Instruments, Chaska, MN, USA) at the laboratories of the Israeli Cattle Breeders’ Association (Caesarea, Israel). This instrument was calibrated monthly by Secondary Reference Material (SRM) produced by Actalia (Poligny, France), and the FA profile in the reference material was determined by GC. The somatic cell counts (SCC) were determined in the same laboratory. Additional milk samples were collected on the same milk collection days; representative daily pools were prepared for each cow according to the milk production for each milking, and frozen at −20 °C, pending the analysis of milk metabolites, G6PDH activity, and indicators of oxidative stress. Postpartum, the cows were fed a standard Israeli milking cow ration. The composition and content of the diet are presented in Moallem et al. [12]. The diet was offered once daily at 10:00 h ad libitum to about 5% orts. The individual amounts offered and the daily leftovers were recorded daily to calculate the individual feed intake.

The EB was calculated according to NRC (2001), as described in Moallem et al. [12] for the first 21 d of lactation, and the cows were divided post-factum into 2 groups. The median of the average EB during the first 21 DIM was calculated as −2.8 Mcal/d. Cows were classified as being in negative EB (NEB, *n* = 19) if the mean EB during the first 21 DIM was less than −2.8 Mcal/d, and as being in positive EB (PEB, *n* = 21) if the mean EB during the first 21 DIM was greater than or equal to −2.8 Mcal/d. Two cows were excluded from the analysis due to extreme EB values. As expected, among the NEB cows, 12 cows (63%) were milked 3 times daily and 7 cows (37%) were milked twice daily; among the PEB cows, 8 cows (38%) were milked 3 times daily and 13 cows (62%) were milked twice daily.

### 2.2. Blood Sampling and Analysis of Metabolites and Stress Biomarkers

Blood samples were taken 3 times weekly (on Sunday, Tuesday, and Thursday) from calving until 21 DIM. After the morning milking, the blood samples were collected by coccygeal venipuncture into vacuum tubes containing lithium heparin (Becton Dickinson System, Cowley, UK), and the tubes were immediately placed in ice. Plasma was separated by centrifugation for 15 min at 1000× *g*, divided into 4 tubes, and stored at −80 °C pending analysis. The concentrations of glucose, non-esterified fatty acids (NEFA), beta hydroxybutyrate (BHB), malondialdehyde (MDA), cortisol, and tumor necrosis factor alpha (TNF-α) were determined. The plasma glucose concentrations were analyzed using the Cobas C111 Autoanalyser (Roche Holding GmbH, Grenzach-Wyhlen, Germany). The concentrations of NEFA in the plasma were determined using a NEFA C Test Kit (Wako Chemicals GmbH, Neuss, Germany). The intra- and interassay coefficients of variation (CV) for the NEFA assay were 5.9 and 6.1%, respectively. The plasma BHB concentration was determined using a RANBUT D-3-Hydroxybutyrate kit (Randox, Crumlin, UK). The intra- and interassay CVs for the BHB assay were 1.3 and 1.6%, respectively. The plasma MDA concentration was measured by the thiobarbituric acid reactive substances (TBARS) fluorometric assay [13]; the intra- and interassay CVs were 9.4% and 2.5%, respectively. The plasma cortisol concentrations were determined by ELISA (EIA1887, DRG International, Inc., Springfield, NJ, USA), and the TNF-α concentration was determined using a bovine TNF-α Duoset ELISA kit (R&D Systems, Inc., Minneapolis, MN); the intra- and interassay CVs were 9.3 and 6.1%, respectively. 

### 2.3. Milk Metabolites and Indicators of Oxidative Stress

To determine the milk metabolites, G6PDH activity, and oxidative stress indices, we randomly selected 12 cows from each milking frequency group. Since the classification according to the calculated EB was done post-factum, the milk analyses were conducted in samples of 13 PEB and 11 NEB cows. Thawed milk samples were centrifuged at 3000× *g* for 20 min at 4 °C to remove the fat layer, and the skim milk was analyzed for milk glucose, G6P, as well as lactic and malic acid concentrations using a fluorometric assay via enzymatic reactions, and the activity of G6PDH in milk was analyzed by modifying a classical enzymatic assay procedure in which the reduction of NADP+ to NADPH is coupled to form a fluorometric chromophore [11]. In addition, the milk MDA concentration was measured according to the TBARS fluorometric assay and the oxygen radical antioxidant capacity (ORAC) in milk serum was analyzed by a fluorometric procedure as described previously [11]. 

### 2.4. Statistical Analysis

Continuous variables (milk, milk solids, DMI, EB, milk, and blood parameters) were analyzed as repeated measurements using the PROC MIXED procedure of SAS, version 9.2 (2002) (SAS Institute, Inc., Cary, NC, USA). When relevant, variables were analyzed using the specific previous lactation data as covariates. 

The model was Y_ijkl_ = µ + T_i_ + L_j_ + C(T × L)_ijk_ +DIM_ijkl_ + *E*_ijklm,_ where µ = the overall mean; T_i_ = the treatment effect, i = 1 to 2; L_j_ = parity, j = 2 or >2; C(T × L)_ijk_ = cow k nested in treatment i and parity j; DIM_ijkl_ = day in milk as a continuous variable; *E*_ijklm_ = random residual.

The interaction treatment × DIM was tested and found to be non-significant; therefore, it was excluded from the model. Autoregressive order 1 was used as a covariance structure in the model because it resulted in the lowest Bayesian information criterion for most of the variables tested. To evaluate the relationship between EB and milk metabolites and FA, a linear regression analysis was performed for each treatment using the REG procedure of SAS (version 9.2). Least square means and adjusted SEM are presented; *p* ≤ 0.05 was accepted as significant.

## 3. Results

### 3.1. Milk Production and Composition, Dry Matter Intake, and Energy Balance

In the present study, the NEB cows produced 10.5% more milk than the PEB cows (*p* = 0.0001; Table 1). The ECM yield tended to be higher in the NEB cows (*p* = 0.06), with no difference between groups in 4% FCM yield (Table 1). No differences were observed in the fat percentage or lactose percentage in milk; however, there was 6.3% more protein in the PEB cow milk than in that of the NEB cows (*p* = 0.0006; Table 1). No differences were found in milk SCC between groups. During the first 21 d postpartum, DMI was 13.4% lower for the NEB cows than for the PEB cows (*p* < 0.0001), and the average calculated EB until 21 DIM was −5.9 and 4.1 Mcal/d for the NEB and PEB cows, respectively (*p* < 0.0001; Table 1). In addition, the DMI from calving until 45 DIM was 7.6% lower in the NEB cows vs. the PEB cows (26.6 vs. 28.9 kg/d, respectively, SEM = 0.3, *p* > 0.0001), and the average calculated EB until 45 DIM was −2.9 and 5.2 Mcal/d for the NEB and PEB cows, respectively (SEM = 0.5, *p* < 0.0001).

### 3.2. Plasma Concentrations of Metabolites and Stress Biomarkers

The average plasma glucose concentration during the first 21 DIM was 9.3% greater in the PEB than in NEB cows (*p* = 0.002), and the average plasma BHB concentration was 23% lower in the PEB vs. NEB cows (*p* < 0.0001; Table 2). The increased BHB and the lower glucose concentrations in the plasma of NEB cows were in agreement with our classification of the cows according to their calculated EB. No differences were observed between groups regarding the plasma concentrations of NEFA, malondialdehyde, cortisol, or TNF-α (Table 2). 

### 3.3. Milk Metabolites and Markers of Oxidative Stress, and the Milk FA Profile

The average concentrations of the parameters examined in the milk are presented in Table 3. The average concentrations of milk glucose, G6P, malic acid, lactic acid, and MDA during weeks 1–7 in lactation did not differ between NEB and PEB cows. However, G6PDH activity was higher in the milk of NEB vs. PEB cows (*p* = 0.03), and the ORAC tended to be higher in NEB vs. PEB milk (*p* = 0.1; Table 3). 

The milk FA profile differed in the NEB and PEB cows; the percentage of C18:1 was higher in the milk of NEB than in the milk of PEB (*p* = 0.001; Table 3); therefore, the total percentage of mono-unsaturated FA (MUFA) was higher in NEB (*p* = 0.01); in addition, the total percentage of unsaturated FA (UFA) was higher in NEB milk than in PEB milk. The percentage of C16:0 tended to be lower in NEB (*p* = 0.10); thus, the total percentage of saturated FA (SFA) tended to be lower in NEB vs. PEB milk (*p* = 0.10; Table 3).

**Figure 1:**
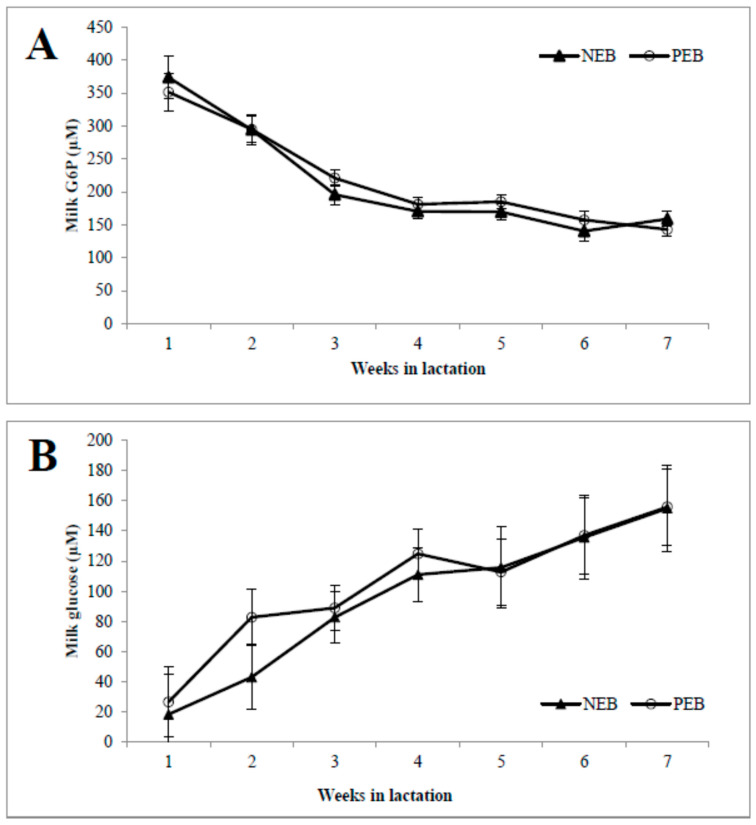

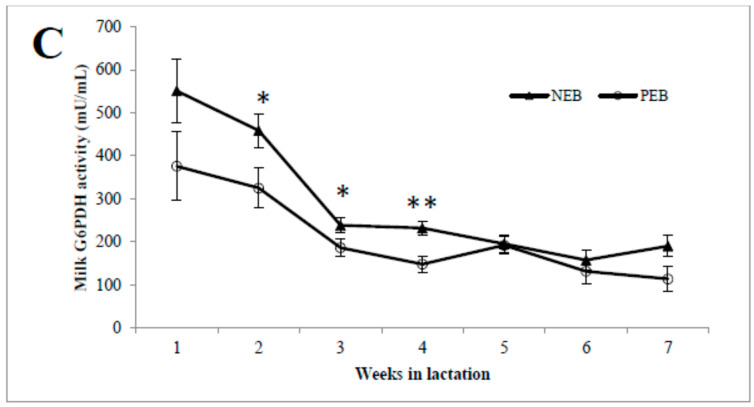
Milk samples from cows that were classified retrospectively as being in negative energy balance (NEB, *n* = 11) or positive energy balance (PEB, *n* = 13), according to their mean energy balance during the first 21 DIM, were examined for their concentrations of milk glucose-6-phosphate (G6P; **A**), and glucose (**B**) as well as G6P dehydrogenase (G6PDH; **C**) activity for 5–45 DIM. * *p* ≤ 0.05; ** *p* ≤ 0.01.

As shown in, the concentrations of milk G6P (Figure 1A) during the first week postpartum were high and then declined until 7 weeks postpartum, and vice versa for the milk glucose concentration (Figure 1B). The activity of G6PDH was highest in week 1 and then declined until 7 weeks postpartum, and it was significantly higher in NEB than in PEB milk in weeks 2, 3, and 4 postpartum (Figure 1C).

### 3.4. Correlations between Milk Metabolites and the FA Profile and the Calculated EB

We tested the correlation between EB and the milk parameters; we found a negative correlation between milk G6PDH activity and the EB (r = −0.39, *p* < 0.0001). The milk G6P and the MDA contents were also significantly correlated with EB, but with small correlation coefficients (Table 4). In addition, the percentage of oleic acid tended to be negatively correlated with EB, and SFA in milk tended to be positively correlated with EB (Table 4). 

## 4. Discussion

A severe magnitude or duration of NEB during the postpartum period raises the risk of metabolic diseases and is associated with reduced conception rates. Milk metabolites and components are desirable candidates to be biomarkers, since this medium is non-invasive and accessible in a commercial setting. Other fluids and tissues, such as hair samples [14], may potentially prove to be a valuable source of non-invasive metabolic biomarkers in the future. Currently, several blood metabolites, the calculated EB, and the body condition score are the traditional methods for detecting NEB; however, they require complex methods such as individual feed intake, body weight, time-consuming blood collection, and the need for trained staff [15,16]. Indeed, in the present study, we showed that plasma BHB levels increased in NEB vs. PEB dairy cows. Elevated blood levels of BHB are linked to an increased risk of infectious illnesses [17,18]. 

Glucose-6-phosphate-dehydrogenase (G6PDH), the first enzyme in the pentose phosphate pathway, has also been proposed as a candidate milk biomarker for NEB diagnosis in dairy cows, based on the relationship between milk G6P and EB. Few studies have examined the presence of G6PDH in cow milk. Zachut et al. [11] demonstrated that milk G6PDH activity in cows peaked during the first and second weeks of lactation before declining until the fifth week of lactation. This is similar to milk G6P concentrations. The patterns of the changes in the concentration of milk G6P and glucose are in agreement with others [10,11]. This supports our earlier hypothesis that elevated G6PDH activity in early lactation reflects increased shunting of G6P to the PPP [11]. However, in this study the average concentrations of G6P in milk did not differ in the milk of NEB vs. PEB; thus, we do not propose it as a candidate biomarker of NEB. On the other hand, there was an increase in G6PDH activity in cow milk with NEB, compared with cow milk with PEB, and together with the correlation between G6PDH activity and EB (r =−0.39), we propose that G6PDH activity can serve as an innovative candidate biomarker for EB in dairy cows for future validation studies, which is in agreement with our premise [6]. As stated above, others found higher concentrations of citrate, cis-aconitate, creatinine, glycine, phosphocreatine, galactose-1-phosphate, glucose-1-phosphate, UDP-N-acetyl-galactosamine, UDP-N-acetyl-glucosamine, and phosphocholine, but lower concentrations of choline, ethanolamine, fucose, N-acetyl-neuraminic acid, N-acetyl-glucosamine, and N-acetyl-galactosamine in milk of cows in NEB in early lactation [7]. Taken together, the strength of our data relies on the premise that various milk metabolites and enzyme activities indicate the cows’ EB during early lactation.

Changes in blood plasma lipidome are evident in postpartum compared with prepartum dairy cows [19]. In milk as well, changes in milk FA composition are known to be related to the metabolic status of dairy cows, because adipose tissue lipolysis during negative EB releases palmitic, stearic, and oleic acids, which are incorporated into the milk fat [20,21,22]. Indeed, an elevated proportion of oleic acid in milk fat during NEB postpartum was demonstrated by Gross et al. [23]. In our experiment, the composition of FA in milk was correlated with the EB of the cows: the ratio of oleic acid in milk was higher in cows with NEB than in cows with PEB. In addition, the proportion of MUFA and UFA was higher, whereas the SFA tended to be lower in cows with NEB. Our results support the relationship between oleic acid and NEB in the milk of postpartum dairy cows, although the correlation only tended toward significance. Moreover, since dietary composition affects milk FA composition and may change the oleic acid percentage in the milk, any changes in the milk FA composition should be interpreted carefully by considering the diet, as previously suggested [22]. Recently, when examining milk metabolites and FA profiles in both a restricted feed model and in postpartum cows, it was demonstrated that milk *cis*-9 C18:1 had a good single linear regression with energy balance, and that milk G6P was negatively correlated with EB [24], which is similar to our findings. 

In conclusion, we found that G6PDH activity in milk is correlated with individual EB in postpartum cows, suggesting that it might serve as a candidate indicator of NEB in postpartum cows, that should be validated in future studies. Studies to determine more novel non-invasive biomarkers of NEB, such as metabolites or enzymatic activity in milk, in dairy cows are warranted.

## Figures and Tables

**Table 1:** Milk production and composition, dry matter intake, and the calculated energy balance of negative energy balance (NEB) and positive energy balance (PEB) cows during the first 45 d in lactation.

	Groups			
	NEB	PEB	SEM ^1^	*p*-Value
Milk yield, kg/d	49.6	44.9	0.9	0.0001
ECM ^1^, kg/d	38.4	36.7	0.6	0.06
FCM ^1^ 4%, kg/d	47.0	45.4	1.2	0.40
Fat, %	4.1	4.2	0.06	0.20
Protein, %	3.2	3.4	0.03	0.0006
Lactose, %	4.9	4.9	0.02	0.30
SCC ^1^, ×10^3^ cells/mL	191.9	173.7	45.3	0.80
Dry matter intake 21 d, kg/d	22.8	26.3	0.3	<0.0001
Calculated energy balance, Mcal/d	−5.9	4.1	0.5	<0.0001

^1^ SEM = standard error mean; ECM = energy corrected milk; FCM = fat corrected milk; SCC = somatic cell count.

**Table 2:** Average concentrations of plasma metabolites and stress biomarkers of negative energy balance (NEB) and positive energy balance (PEB) cows during the first 21 d in lactation.

	Groups			
	NEB	PEB	SEM ^1^	*p*-Value
Glucose, mg/dL	57.5	62.9	1.1	0.002
BHBA ^1^, mg/dL	7.8	6.0	0.27	<0.0001
NEFA ^1^, µEq/L	459.8	392.2	33.0	0.17
Malondialdehyde, µM	323.1	301.5	0.69	0.82
Cortisol, pg/mL	8.6	7.7	0.9	0.59
TNF-α ^1^, pg/mL	339.8	402.2	33.0	0.25

^1^ SEM = standard error mean; BHBA = beta-hydroxybutyrate; NEFA = non-esterified fatty acids; TNF-α = tumor necrosis factor alpha.

**Table 3:** The average concentrations of metabolites, oxidative stress markers, and the FA composition in the milk of negative energy balance (NEB) and positive energy balance (PEB) cows during the first 45 d in lactation.

	Groups			
	NEB	PEB	SEM ^1^	*p*-Value
n	11	13		
Glucose, µM	88.3	108.3	13.8	0.30
Glucose-6-phosphate, µM	224.5	229.1	12.3	0.80
G6PDH activity, mU/mL ^1^	323.0	229.0	28.7	0.03
Malic acid, µM	347.2	323.7	17.4	0.30
Lactic acid, µM	237.7	231.8	21.7	0.80
Malondialdehyde, µM	212.3	338.7	123.8	0.30
ORAC, µM ^1^	217.0	206.3	4.5	0.10
Milk fatty acids, %				
C16:0	1.08	1.14	0.03	0.10
C18:0	0.39	0.40	0.01	0.32
C18:1	0.98	0.85	0.02	0.001
SFA^1^	2.57	2.70	0.06	0.10
MUFA ^1^	1.31	1.18	0.03	0.01
PUFA ^1^	0.24	0.22	0.01	0.30
UFA ^1^	1.51	1.38	0.04	0.02

^1^ SEM = standard error mean; G6DPH = glucose-6-phosphate dehydrogenase; ORAC = oxygen radical antioxidant capacity; SFA = saturated fatty acids; MUFA = monounsaturated fatty acids; PUFA = polyunsaturated fatty acids; UFA = unsaturated fatty acids.

**Table 4:** Correlations between energy balance (EB) and milk metabolites, oxidative stress markers, and FA composition during the first 45 d in lactation.

Correlation to EB	r	*p*-Value
Glucose	−0.02	0.36
Glucose−6-phosphate	−0.18	0.008
G6PDH ^1^ activity	−0.39	<0.0001
Malic acid	−0.13	0.08
Lactic acid	0.03	0.65
Malondialdehyde	0.17	0.02
ORAC ^1^	0.03	0.73
C16:0	0.14	0.24
C18:0	0.16	0.17
C18:1	−0.20	0.08
SFA ^1^	0.21	0.07
MUFA ^1^	−0.11	0.33
PUFA ^1^	−0.06	0.61
UFA ^1^	−0.09	0.43

^1^ G6DPH = glucose-6-phosphate dehydrogenase; ORAC = oxygen radical antioxidant capacity; SFA = saturated fatty acids; MUFA = monounsaturated fatty acids; PUFA = polyunsaturated fatty acids; UFA = unsaturated fatty acids.

## Data Availability

Not applicable.
